# “Ripping off the band-aid”: uncovering future health care professionals' “fractured knowledge” about sexual and reproductive health

**DOI:** 10.3389/frph.2024.1242885

**Published:** 2024-03-25

**Authors:** Angie Mejia, Kara Nyhus, Tessie Burley, Alexis Myhre, Marcela Montes, Kristin Osiecki, Anita C. Randolph

**Affiliations:** ^1^ Community Engagement and Education (CEEd) Hub, Masonic Institute for the Developing Brain (MIDB), University of Minnesota Twin Cities, Minneapolis, MN, United States; ^2^ Center for Learning Innovation, University of Minnesota Rochester, Rochester, MN, United States; ^3^ School of Nursing, University of Minnesota Twin Cities, Minneapolis, MN, United States; ^4^ Augsburg University, Minneapolis, MN, United States; ^5^ Center for Health Equity, Minnesota Department of Health, Minneapolis, MN, United States; ^6^ Department of Paediatrics, University of Minnesota Twin Cities, Minneapolis, MN, United States

**Keywords:** undergraduate students, sexual health education, SRH (sexual and reproductive health), young adults, health sciences, healthcare profession students

## Abstract

**Background:**

Research has shown the role of identity on future health professionals' confidence and competence in addressing the sexual and reproductive health (SRH) needs of their patients. While there has been some work in increasing the sexual health literacy of future providers via various curricular approaches and comprehensive clinical-based training, there are research gaps on how social differences around identity impact future healthcare professionals’ knowledge and practices around SRH.

**Objectives:**

This article presents research findings on the experiences of US undergraduate students attending a campus that provides training in the health sciences and health professions. Our study aims to understand the perspectives of these students as they pertain to their future career choices in healthcare, with a focus on how their past experiences learning about sex, sexuality, and reproduction impact their current and future professional trajectories.

**Methods:**

We present a qualitative analysis from 40 in-depth interviews with U.S. undergraduates. The interview questions were designed in collaboration with undergraduate researchers interested in sexual health education. These student researchers collected all the interview data and worked with senior researchers to analyze some of these data.

**Results:**

The themes that emerged from the interviews were around experiences with what students perceived as “fractured” sexual and reproductive health (SRH) knowledge they received as children and adolescents. This knowledge shaped essential aspects of their identity as young adults and future healers. Data indicated unique processes implicated in how past as well as present socialization experiences learning about sex, sexuality, and reproduction positions undergraduates in health professions to see young adulthood as a journey of “catching up” on sexual knowledge but also as an ongoing experience of anticipation and planning influencing their career-building journey.

**Conclusions:**

The importance of sexual health literacy among healthcare professionals cannot be overstated, as it is vital in providing patient-centered and non-judgmental sexual and reproductive health (SRH) care and services. To date, there is a shortage of studies looking at the impact of sexual health knowledge on healthcare professionals. More research is needed on educational strategies that could be implemented at the intra-personal level to assist college-aged young adults in healthcare career tracks to “catch up” or “fill in the gaps” in their sexual education journey.

## Introduction

Increasing health professionals' sexual health literacy is vital to developing better curricula, improving clinical training, and solidifying practices for providing sexual health care in a patient-centered and non-judgmental manner. Research points to various factors that might impact how students in healthcare training programs address issues around sexual and reproductive health (SRH) with their future patients. Many of these studies focus on how curriculum- and clinical-based training on sex and sexualities increases future health professionals' levels of comfort, competence, and confidence in addressing SRH with patients (1–5), which in turn decreases barriers to care in populations, especially those currently considered underserved by health services (6–8). However, most of this research into the experiences, knowledge, and understandings about sex and sexual health impact how health professions students learn to deliver this sexual health care does not take into consideration how identity-based markers of social difference shape future healthcare professionals knowledge, practices, and competencies around SRH (9, 10).

Foundational knowledge about sex, sexuality, and similarly adjacent health matters is of critical importance for U.S. young people seeking careers in healthcare (1, 11). Despite university environments presenting young people the opportunity to increase their health literacy and exercise self-determination and autonomy over their health needs and practices (12, 13), as well as provide them with a broader range of resources and services (14), this age cohort bears the disproportional burden of sexual health inequities and disparities (15–18). In addition**,** even when many in this group might be embedded in educational contexts where the intersections between sexuality and health might be part of the curricula, research has found that future healthcare providers have low levels of sexual health literacy (1, 19–21), hold myths about sex, sexuality, and reproductive health (22), vary in their understandings of how reproductive health policy impacts their career choices (23), engage in unsafe sexual health behaviors (19, 24, 25), and experience trouble access treatment for sexual health issues (26–28).

To our knowledge, no in-depth studies focus on the lived experiences of health profession students learning about SRH before adulthood and how these experiences might shape their career trajectories, aspirations, and future clinical practices. This article contributes to this scholarly body of knowledge by providing findings on the sexual health education experiences and needs of post-secondary students attending healthcare, health sciences, or health professions programs in the U.S. We present a qualitative analysis from 40 in-depth interviews with U.S. undergraduates attending a small campus offering training in the health sciences with avenues for further training in the health professions. We found that this group of youth perceives the entrance to adulthood and engages in planning for healthcare careers without the right tools, knowledge, and range of possible perspectives about sex and sexuality. We argue that this group's collective anxiety of needing “to catch up” or “fill in the gaps” in their existing “funds of knowledge” (29) around sexual and reproductive health is inherently connected to multiple identity-meaning processes in their lives as emerging adults.

## Literature review

Knowing how essential identity factors, such as gender, ethnicity, class, age cohort, religious practices and identifications, and parental educational backgrounds, among others, and the experiences that emerge from the intersectional nuances that shape these, impact healthcare providers as well as those training to become one (30), there still a lack of research studies examining the way the personal attitudes, experiences, and perspectives of future healthcare professionals shape how they will address the sexual healthcare needs of patients in the future (2, 31). Gender, for example, has been connected to levels of comfort during learning of sexual health-related matters (32), as well as empathy and the impact it has on the patient-provider relationship (33, 34). Hojat et al. study showed how students identifying as female scored higher on the Jefferson Scale of Physician Empathy scale than those identifying as male, in which empathy scores were associated with higher rates of clinical competence skills demonstrated by women than men (35). One's gendered identity and behaviors connected to embodying said identity are related to students' level of comfort when talking about sex with patients. Research has found that subscription to more traditional ideologies around masculinity detracted from male-identifying students' ability to feel comfortable discussing sexual health issues with their patients (3).

Religiosity (36, 37), ethnic background (24), and levels of acculturation (38) may also impact how students in the health professions engage the sexual needs of future patients in their care (31, 39). Among nursing and medical students, having not had sex, religion, and political ideology were demographic variables connected to negative attitudes toward sexuality, especially those behaviors, practices, or identities considered taboo and socially controversial (40). A survey of medical students showed that those self-identifying as having sexual experiences felt more comfortable conducting gynecological exams than those who did not (41). Other research indicates that students' subscription to prejudicial and stereotypical ideas about sexuality might impact how they deliver care to members of socially stigmatized groups such as sexual minorities (24, 42), sex workers (43, 44), older adults (45), and patients with disabilities (46), including those with intellectual disabilities (47). Factors such as self-reported level of sexual experiences, presence of sexual health conditions, and perception of the sexual health training received during their training shaped how comfortable medical students would feel addressing and caring for future patients' sexual health concerns (21). Personal choice of contraceptive type, for example, impacts how healthcare providers counsel patients seeking advice on family planning issues and other sexual health concerns (48). Similarly, a study on contraceptive use patterns by medical students posits that those who practice safe sex behaviors are more likely to feel comfortable addressing safer sex practices with patients in the future (49).

Though there is a small but growing number of studies analyzing perspectives and attitudes shaping how practitioners provide sexual health care to patients, there is not much research on the role of past personal experiences or what West et al. (10) refer to as early socialization factors. These identity and socialization factors, at the institutional level of influence (such as knowledge received in educational, religious, or via other institutional bodies), interpersonal contexts (instruction delivered by family, trusted adults, or friends), and socio-cultural ones (such as access to knowledge via media) shape how beliefs, practices, and behaviors around SRH identity formation and future practices. To our knowledge, no in-depth studies examining the role of personal experiences and socialization on sexual health matters in undergraduates training for healthcare careers. Thus, we look to studies on young adult populations to elucidate the role of previous sexual health knowledge provision on current social practices and behaviors. Research has shown that parental and extended family members' communication with adolescents about sex is associated with the latter's use of condoms (50–52) and their increased likelihood of discussing safe sex practices with their intimate partner (17, 53). Despite research showing that sexual health education delivered by parents and other trusted adults may have a positive influence on young adults' sexual health beliefs, choices, and practices (51, 54–56), the latter group still perceives the SRH knowledge received as children and adolescents not meeting their needs (50, 51, 57–59). Similar sentiments are reported in studies focusing on young adults' perceptions of and experiences with sexual health education and promotion in K-12 contexts (57, 60, 61) and during their undergraduate college years (12, 62–64).

While there might be an assumption that students enrolled in health professions programs may have a more nuanced understanding of the intersections between sexuality and health due to its centrality to their work with patients, healthcare professionals, as well as those in health professions programs, might not be much different from the populations they serve. For instance, medical students' definitions of what practices and behaviors can be attributed to “having sex” mirror the understandings held by other university student populations (65). Healthcare providers do engage in risky sexual practices (11, 25, 66) and endorse myths about sex, sexuality, and same-sex practices. Their understandings and perspectives towards those with gender variant identities are also diverse (8, 9, 20, 31). This group also reports varied levels of sexual health literacy (19, 67, 68), even when some of those perspectives reflect those of young adults growing up in countries with comprehensive sex education schemas (69).

In light of these gaps in the literature, we present an in-depth examination of the experiences of health professions undergraduates and the impact sexual health knowledge and instruction previously received as children and adolescents have on their identity and career aspirations as future healthcare professionals. Expanding on the possible processes between sexual health knowledge acquired before young adulthood and various dimensions and contexts of identity formation informs our future practices in providing better sexual health education in the U.S.

## Methods

Data collection for this article was undertaken as part of a larger mixed-method and community-based participatory research study on the sexual and reproductive health needs, perspectives, and beliefs of undergraduate students enrolled in programs offering training in the health and medical sciences. We present our analysis of various forms of qualitative data gathered from June 2022 until the present: In-depth interviews collected by AM, MM, KN, and TB, ethnographic participant observation notes of community and other public events kept by AMM, KN, and KO. More analysis of survey data collected as part of this larger study is reported elsewhere (70). This study has been approved by the University of Minnesota's Institutional Review Board (IRB) as protocol # 14301.

### Participants

We conducted a total of 40 in-depth interviews. These interviews took place in Southeast Minnesota, a US state located in the Midwest, beginning in June 2022, with one-third being of these being completed in 2023. 87.5% of our sample self-identified as female assigned at birth, 7.5% of participants self-identified as male assigned at birth, and 5% self-identified as gender non-binary. 44% of our sample identified as LGBTQIA+. Almost half of our total sample (47%) identified as Black, Indigenous, or other People of Color (BIPOC) or multiracial. 26% of those interviewees mentioned being raised in a religious household. Our interview participants partly mirror the campus' demographics: 70% of female-presenting individuals comprise most of the Health Science University (HSU's) student body. As a minority-enrolling university, 40% of HSU's students self-identify as people of color, and over 80% identify as first-generation college students. We applied the methodological wisdom of feminist researchers by having student undergraduate researchers (and not the senior researchers in this paper) conduct all interviews. As Bhopal (71, 72) and others have shown, shared identity between respondent and researcher allows for a more nuanced understanding of the phenomena being explored (73, 74). We believe that having someone like them as interviewers ensured participants' comfort in discussing topics that may be embarrassing or stigmatizing if discussed with someone who was not a near-aged peer. To assure further reflexivity, those interviewers met with KN to debrief and discuss any issues that may have arisen during the interviews.

### Analysis

Findings emerged from a thematic analysis of interviews conducted beginning February 2022 until the present time. To analyze these data, KN and AMM created a preliminary codebook using codes that emerged from past codes from AMM's previous research on students' perspectives on the impact of U.S. reproductive health policy on their careers (23) as well as a sustained engagement with the literature on sexual health education. Using these codes, KN and AMM coded 1/14 of the interviews. KN and AMM met twice to check their coding and finalize a complete codebook with categories. They then coded all transcripts (first coding pass) before asking one of AMM's colleagues, trained in qualitative research methods, to code transcripts to assure intercoder reliability. On the second coding pass, AMM drafted memos and summaries of the themes that emerged from this categorical analysis and discussed these with KN and a small group of students interested in sexual health and reproductive justice. Conversations with this group allowed AMM, KO, and KN to get feedback on the themes and patterns they summarized into findings when drafting an earlier version of this article. KN and AMM looked at the categories and engaged in another round of analysis when submitting changes to this manuscript. Ethnographic observations from activities around sexual health promotion and similarly connected educational events on and off campus were kept by AMM, KO, and KN and are used in this article to provide further context.

### Institutional background

Our participants, at the time of analysis, were all students currently enrolled in Health Sciences Innovation (HSI), a campus in the Midwest with the educational mission to prepare undergraduate students to enter health and medical sciences careers or post-baccalaureate programs in the healthcare professions. Due to being a relatively young campus, HSI does not run a student health clinic on-site and relies on the health services provided by a local clinic. This clinic provides students with some preventive-level sexual and reproductive care services, including prescriptions for hormonal contraception, condoms, pelvic exams, and gonorrhea and chlamydia testing. If students need other services, the clinic provides them with referrals to other providers. More on the quantitative findings of HSI student uses of existing sexual health care services are available elsewhere (75).

## Findings

We sought to answer the following questions: How do undergraduates in a health professions program make sense of previous experiences receiving sexual health knowledge and resources from schools or parents? How do these sex and sexuality “funds of knowledge” impact these students' experiences as emerging young adults? And what do their past experiences learning about sex, along with their knowledge of becoming educated and better informed on sexual health matters, inform their unique sexual health literacy needs? This section will expand on three themes that emerged from our continued analysis of interview data: First, we found that experiences receiving sexual and reproductive health education, knowledge, or resources from adults in their lives, be they school-based or family, were perceived in a critical light and seen as having been incomplete and at times, detrimental to them as adolescents. Second, these gaps left them with a need to “catch up” on their understanding of sex, sexuality, and sexual health, which impacts how they navigate their lives as emerging adults. Third, having to “catch up” from knowledge deficits from the past combined with their experiences accessing and receiving sexual health services as young adults shape desired practices and skills they wish to master as they enter future healthcare careers.

### “I wished they would have … ”: perspectives on sexual health instruction by parents and K-12 schools

While our respondents indicated that both sources of information—parents and schools—did not measure up to their minimal standards, there were differences between them. For many of our participants, their recollections reflected parents lacking the training, knowledge base, talents, or skills to provide them with a comprehensive overview of their sexual and reproductive health needs.

One thing that always bothers me with sexual health is that no one talks about puberty in the way I think they should. Because puberty is a big part of sexual health and how this all blossoms into being important. So even things like hygiene aren't really ever covered. It's an assumption that parents will fill in that gap. But again, it's one of those things where parents don't always know the answer. So how do you properly clean yourself before and after sex? Nobody really talks about that, and sometimes, parents don't know the answer either. So things like hygiene don't really get covered well enough[.]

Some of the ways parents delivered education about sex and sexuality were not perceived as altogether wrong by our participants. Instead, this mode of providing information about puberty and sexuality was a product of parents “not knowing” what was necessary for their children to learn about these topics. Below, a participant shares her reaction when receiving the *American Girl: Caring and Keeping of You* booklet, a popular sexual education booklet from a well-known doll manufacturing company in the U.S.

…this [booklet] is boring, like where is the drama. I was like, “this isn’t interesting”. I don't want to read this and just like, hid it under my bed.

Even when their parents sought to provide knowledge on biological changes experienced during puberty, this participant, like some others, may have wanted to learn about “the drama”, which we interpreted as those socio-emotional complexities connected to growing up as a sexual being in adolescence.

Except for three participants who attended high school settings with curricula aligned with U.S. comprehensive sexual health education standards or innovative health promotion practices (for instance, one participant indicated having attended a high school that had hosted a sexual health fair for their students), many participants viewed the sexual and reproductive health education they received in educational K-12 settings negatively.

I took my health class in my freshman year during second semester, and our textbook … was so old that it was barely colored picture.

We got very little in detail about stuff in middle school, but I didn't learn the names of anything until freshman year of high or eighth grade, freshman year of high school. And I started looking into stuff like what do I have? What is all that down there? And I had to do my own research because I wasn't taught of those things were, I knew practically nothing because they just didn't teach us any anatomical term … And that was just not as in-depth as I wish I would've known about myself going into, especially high school and a public high school[.]

Most of our interviewees had a critical view (with a few showing a strong distaste) of the approaches some educators in school settings used to deliver sexual health education to their peers. Some indicated that their schools' methods and choice of material created stigma, fear, and anxiety.

I remember they brought in this doctor and they put all of us in the theater and showed a bunch of disgusting pictures of STDs to scare us. … I feel like it did more harm than good because it stigmatized those different illnesses. Because after seeing that every, all these young students, they're like, that's gross I'm never going to have that. So, I think it honestly did more harm than good … It was like a whole day dedicated to looking at these STD pictures. I wish they didn't bring our entire grade into the auditorium just to show these pictures I feel like they could have shown them in health class when we're actually learning about it rather than making this big grim spectacle of it.

Participants disapproved of the approaches used to impart these lessons and the accuracy of the information provided by school teachers, staff, and, in the segment below, guest speakers.

And outside of just this one class, we had a speaker that came in and talked to us about purity culture. And that was just as bad if not worse. I was on birth control at the time because I had some pretty bad acne. And some pretty bad skin disorders that I still have now and we were using birth control as a method to try to treat this. And I remember sitting in this auditorium listening to this guy talk about birth control and he basically, told us that it was plan B, which it's not. And I got like the worst dread of my life because up until this point I had basically, only been taught that killing or abortion was killing a baby. And if I was using plan B then I was basically getting an abortion. And I should feel terrible about myself for trying to treat myself for a medical disorder. And it was like I was looking back on it, completely ridiculous and completely untrue. Especially if you’re telling someone who is, I think I was 14 at the time.

This participant's experience receiving instruction from a guest speaker at school shows us not only did the information make her feel “terrible” for choosing to use birth control for a common medical condition in adolescence (acne), but the information delivered about contraceptives such as Plan B, was erroneous.

### Filling the void: catching up on sex-ed as a young adult

Throughout our analysis of interviews, as well as our ethnographic observations into how students in HSI accessed sexual health, there was a salient theme of needing to “catch up.” This prevailing theme was almost a shared sentiment our participants held about entering adulthood. “Adulting” was also in experiencing the rituals of “catching up” on the knowledge they did not receive or having to “fill in the blanks” left by the sexual health knowledge they received (or failed to) receive as children and adolescents.

Several of our interviewees felt that those past lessons connected to sexual and reproductive health needed to incorporate content on the link between self-care and sexual health. Like many participants, the respondent below focuses on their spotty understanding of daily rituals connected to hygiene and other practices related to having sex in ways that prevent further health conditions such as UTIs.

And like, there's like ways to take care of yourself before and after sex that wasn't ever really discussed. …. Like something I found out recently, you pee before and after sex. That is literally not something that they teach you in sex ed.

In addition, there appeared to be a level of anxiety connected to the health care practices that one would need to receive when becoming an adult.

[P]eople don't really tell you that once you turn 21, your doctor's going to start telling you, “it's time to get a pap smear”. Nobody really prepares you for this. I just keep wondering, okay, what's next? What are these things, like hormonal changes? Nobody really tells you how that plays out. So sometimes I get confused with that. It's like, how do you know what's right? How do you know that everything's kosher if you will? How do you know considering where you are in life? So I think sometimes I get confused about that. It's like, am I on track? Is there a timeline progression of how things are supposed to be going?

Several respondents indicated that “catching up” was more than just filling in the gaps left by family or school or re-educating themselves on erroneous information received in the past. Catching up was also about wanting a deeper understanding of the socio-emotional complexities implicated in intimate relationships and sexual encounters that were connected to being a young adult.

I feel that there's always more to learn and my teacher did touch on the fact of dating, dating more people. But I felt she could have gone more into the concept of why, how everybody has their own preferences or have their own body, their own mindset on how things work. And having those discussions, I think we thought more of the biological viewpoints than socially and the way that our mind works when thinking about these topics and how we communicate with each other about these types of topics.

I don't think I was prepared for like shitty men. I wasn't prepared for the fact that people will pretend to like you … and that's all they wanted. And so, I had a big stretch of sophomore year where that happened multiple times. And I feel like if I could go back, just with that knowledge that would have changed a lot, I think … looking back on it, it was clear that these boys were not looking for relationships, were not looking for anything more than physical[.]

Catching up or filling in these knowledge blanks meant taking their education into their own hands.

I guess I turn to reading more in general about female and male anatomy, both just in depth. And then more YouTube videos maybe. I don't have a specific person or anybody I know of that I could go to, so I guess just resorting to my roots of Google searches and YouTube videos.

As another participant shared with us, seeking knowledge about sexuality and sex on your own was not something altogether helpful and may have negative impacts on future sexual practices.

I had accidentally stumble on to like fan fiction at a young age so I feel like that kind of clouded my judgement because I was like wow! No thanks.

### Developing a sexual health knowledge baseline: sexual health knowledge for future healthcare careers

Due to the institutional environment that defined the socio-academic day-to-day of our participants, we asked them to reflect on the role SRH has on their future healthcare and medical science careers. When responding to the prompt “What would you have done differently in your learning experience about sexual and reproductive health?” participants indicated frustration that these gaps in sexual health education persisted in the present.

I have noticed that there are a lot of heteronormative approaches[,] not only in healthcare[,] but specifically in sexual and reproductive education. So it's really frustrating because the approach is not versatile, and then there's not a lot of other resources as well to be referred to.

We also found out that their experiences as healthcare consumers accessing and receiving sexual health services impacted their desires for the type of professional skills they wished to embody while providing their future patients with care.

I feel like I would just want to replicate that [experience at Planned Parenthood] with other patients because they made me feel so comfortable and welcomed, and like I didn't have anything to hide. That's just what I would want to do for patients, as well when it comes to whether it be the information I'm giving them, the tone I'm using, the topics I bring up, or even if it's just about any plans throughout the day, kind of thing. Something casual. I feel like I'd want to use that.

Even though there were not as numerous, positive experiences receiving sexual health care services provided to our participants served as examples of practices that they would want to replicate with patients in the future. However, there were more instances of negative healthcare encounters in our interviewees' recollections of sexual health services received as young adults.

I was having my IUD placed last year … [The provider] asked if I wanted to be STD tested right away while she was inserting my IUD and I was like, no, it's fine, I was just tested a few months ago at my annual physical and I only have one partner and we're monogamous so I'm not concerned about it and she still swabbed me and ran the test even after I told her no. So, I think that's my worst experience related to sexual and reproductive health … Yes, I think it just made me more respectful of other people's decisions and realizing like, well you may not agree with them, but it's still their body and their choice.

Just like the above participant's reflection, others felt that their encounters with healthcare practitioners providing sexual and reproductive health care were disempowering experiences that provided them with models of what not to do in the future.

Students continued to surprise us with their insight into what a provider should do and know when they enter careers in healthcare, even when this trajectory did not include addressing patients’ sexual and reproductive health needs of patients. For instance, a participant planning to work as a social worker in a hospital setting indicated which topics were critical for someone in this position to be informed about.

I really think that like sexual assault, rape, LGBTQ reproductive rights, and women's reproductive rights are big ones because … if handled improperly, can have detrimental effects like suicide … And I think that anyone who doesn't go into that kind of profession expecting to deal with that is lying to themselves …

Even when interested in a neuroscience career, the student below emphasized the need for a “good baseline” of sexual and reproductive health knowledge. These students felt this knowledge was necessary as younger people become more informed about sex and sexuality.

The students and the children of tomorrow's world are only going to get more and more educated about these topics. So I would want to have a good baseline to help people get a good understanding of like, what is sex? When can you have sex? Why do you have sex? Like, is it okay to not want to have sex? Is it okay to want to have a lot of sex? Just bringing things up to that perspective of getting the word out there, if that makes sense, is what I would want to do in my future career.

Some of our participants have already worked in healthcare settings while completing coursework towards an interdisciplinary bachelor's degree focused on health and medical sciences. One participant, as we will show, did not learn about sexual health in their native language, which would cause difficulty communicating some anatomical information to patients who speak Arabic. In this situation, the student reflects on how she is explaining to their patient how to collect a urine specimen: “Use both wipes to clean your penis before beginning. Start urinating in the toilet before collecting in the cup”. The student learned most of her knowledge on sexual and reproductive health in Arabic but still did not know the right words to use when explaining a basic procedure to a patient.

You don't want to be in front of your patient looking around feeling shy. And I feel shy or embarrassed, not because I'm shy of the topic that I'm describing, but rather just like, “What if I don’t deliver this to the best of my ability? Or what if I deliver this in a casual setting when this person is looking up to me as a provider?”

We saw many instances of HSI students working in entry-level healthcare jobs or engaging in experiential learning activities within clinical settings as volunteers, where they might not have the needed training on sexual and reproductive issues to do their jobs. Thus, participants may have had to rely on their research when encountering a patient.

I am always surprised how many sexual assaults come into the ED … So, it was like, what do they do with patients who come in? So, I definitely did some research on that. I am always surprised by how … often they pop up.

The quote below is from a student working as a personal care associate (PCA), an entry-level but vital patient-facing role in many US clinical settings. This participant described how she had enough knowledge to understand that a patient with a catheter had something interfering with their genitals. Her experience made her an outlier because she felt comfortable discussing this issue with their patient. However, not all PCAs would be able to do this, based on their previous knowledge of sexual health and what we know from the research on healthcare professionals' sexual health literacy levels.

After you have a catheter, and it has been removed, some people experience bladder leakage. Explaining that you might need to wear a pad or a brief for a few days, that's normal. Validating that that's normal.

Unlike this participant, several indicated feeling unprepared to deal with questions related to sexuality and reproduction, which we learned were anxieties held by those going into fields where sexual health matters would appear. These matters were not part of the job description nor their training.

Sexual and reproductive health knowledge in their future careers was a matter of anticipated importance for our respondents. They all had intended careers across the board, ranging from gynecology to prosthetics, medical social work to sonography. In many of these occupations, sexual and reproductive health training may not be included in the curriculum of study planned for that specific profession or part of the clinical training students may receive after completing coursework. Yet, our students identified numerous ways that they could see SRH issues showing up once they entered their intended careers. Table 1 lists how students anticipated sexual and reproductive health issues in their future careers and the importance of SRH to professionals working or soon-to-be working in those careers. Expanded examples of participants' anticipated knowledge and training needs about careers on this table are not meant to be comprehensive. Still, they might provide insight into the expected SRH skills that these future health professionals connect to their intended career choice.

**Table 1:** Health sciences students' anticipatory sexual and reproductive health knowledge needs.

Health career focus	Importance of sexual and reproductive health knowledge
Reproductive organ specialties (e.g., obstetrics, gynecology)	Nearly all these specialties rely on sexual and reproductive health knowledge
Critical organ specialties (e.g., neurology, cardiology)	Understandings of the proper functioning of critical organs during sexual activity and reproduction must be understood by these providers
Accessory organ specialties (e.g., dermatology, dentistry, prosthetics)	Understandings of potential limitations that medications or procedures could have on sexual and reproductive activity should be understood, as well as how to recognize signs of sexual abuse via accessory organ inconsistencies
Psycho-Social specialties (e.g., psychology, social work)	Knowledge of the effects of hormones, sexual desires, and relationships on an individual, as well the negative consequences that may arise from these, as well as coercion or abuse
Misconduct specialties (e.g., emergency department, forensic pathology)	Recognition of sexual assault on a patient's body and ability to distinguish between natural reproductive processes and unnatural harm
Technical specialties (e.g., radiography, sonography, surgical assistant)	Recognition of foreign/contraceptive items, diseased anatomy, or pregnancy on imaging and in the surgical field
Hospital stay related specialties (e.g., nursing, intensive care unit)	Recognition of the effect of life-saving or long-term care on urinary/reproductive anatomy

Table 1 broadly highlights student-identified interconnectedness between their aspired careers and the expectations of how sexual and reproductive health knowledge could be helpful in the corresponding occupations. Each of the specialties included in the table was an intended career path and anticipated sexual health issues they would encounter, as mentioned by students in our study. While each career has a unique impact on and by the sexual and reproductive health system, the table above categorizes these specialties into groups based on their general interconnectedness with sexual and reproductive health. Regardless of our students' intended future careers, most recognize at least one way that sexual and reproductive health knowledge would be pertinent in the path of study or profession they are interested in pursuing.

### What can be done now to start filling the gap?

In one of the events observed as part of the ethnographic activities connected to this study (an undergraduate research presentation hosted by *Planned Parenthood*), an audience member shared how her generation needed a “big reset”. In her words, “people [her] age” need to “dump and forget” all of the misinformation received from their schools' sexual education programming. This response vibed with most of the audience members, and in a way, it also reflects our participants' feelings about the SRH knowledge, instruction, and resources received in the past. Further, they would most likely agree with this attendee that previous knowledge about sex and sexuality may not only be unusable, but it may have also plagued them with self-doubt about the best way to navigate their sexual lives, whether it was via their health, their relationships, or sexual practices, or even their future careers.

Students who took part in our study offered various recommendations for helping young professionals “catch up” on sexual and reproductive health education. One student, reflecting on her gaps in knowledge as she moves into a career in healthcare, reflected on the importance of required sexuality coursework during undergraduate.

I feel like a college class is a very unbiased way of learning information, and it's also the easiest and most accessible to us. It's not the only way, but like, we're college student[s], just sign it up, and then you're good to go … And like, you're never too old to learn about sex … It's not just, we're like prepubescent kids, you know? There are still like 25 year old men who don't know how a period works. So I think it's just beneficial for everyone to just learn about.

This student points out that there is always room to enhance one's understanding of sexual and reproductive health topics, regardless of age or professional status. We have compiled the rest of our participants' recommendations on improving sexual and reproductive health for youth in Table 2.

**Table 2:** Future implementations to improve sexual health curriculum for undergraduates.

Solution	Proposal
Standardize Sex-ed Content	Require universities to offer sexual education courses connected to graduation requirements to allow students from various backgrounds to “catch up” and receive a standardized presentation of sexual and reproductive health topics.
Expand Sex-ed Content	Cover content in courses that can be then expanded and modified to include information that is relevant to student-reported needs and curiosities.
Access to Information	Develop sexual and reproductive health resources that are easy to understand and locate. These resources should be jargon-free, safe, and available to all students.
Honesty, Depth, and Nuance	Provide accurate and sufficiently detailed information about sexual health, delivered in an age-appropriate manner.
Comfortable Environments	Create sexual and reproductive health educational settings that are physically and emotionally comfortable, with expectations of maturity, to foster trust during vulnerable conversations.
No “Scare Tactics”	Teach safe and healthy sexual behaviors without using fear as a mechanism to convey potential consequences; instead, the implementation of a neutral or neutral-positive delivery of information could help filter out fear-mongering language.
Sufficient Student Support	Establish a standard of support for students, regardless of the personal decisions they make, could create an open and enriched learning environment.

Students' pearls of wisdom from their own lived experience on how to help current young adults like themselves “catch up” on valuable sexual and reproductive health information and improve sexual health curriculum for future students apply to both K-12 and post-secondary settings. The suggestions in Table 2 were summarized from interviewees' criticisms of their sexual health education received in the past, specific understandings of what was needed for them to fill their gaps, or expressed desires on what they wish could have occurred when they had to learn about sexuality. Our work as community-engaged faculty, graduate students in health professions, health services practitioners, and scholars includes ongoing support for a student-led sexual health promotion effort. Details of this intervention and preliminary findings of access to sexual health services are explained elsewhere (75).

## Discussion

Seeing that 38% of young adults aim for future careers in healthcare (76), there is an urgent need to understand how their professional identities are shaped by what they see as their health literacy gaps and deficits and how they wish to repair the fractured knowledge received before entering adulthood. We showed that our participants' experiences receiving and attempting to navigate their sexual lives with inadequate amounts of sexual health literacy were linked to aspects of their identities as emerging young adults but also as future professionals using anticipation to engage in their sexual education. We argue that this dynamic between previous SRH knowledge and lessons they must relearn or renegotiate as they “struggle to adult” are also aspects of anticipatory anxiety about their future roles as healthcare providers.

One of the central findings emerging from our participants' narratives was a clear sense of needing to “catch up” on knowledge around sex, reproduction, and intimate and romantic relationships. This worry over what one should have learned as a child, at least in our unique undergraduate sample, might be connected to various factors. For instance, Goldfarb et al.'s recent work on young adults' recollections of sexual health messages received at home suggested that the latter read parental silence on questions about sexuality and sex as a “sign of parental discomfort or disapproval” (59). We link youths' anxiety over interpretations of how older adults in their lives react when asked for guidance on these matters to recent scholarly work showing how societal anxiety, broadly speaking, might be a uniquely situated experience in this cohort of college-aged people (77). In a qualitative exploration of U.S. women attending college, Talbot (78) found that anxiety connected to “adulting” (that is, the ability to navigate day-to-day life as an adult) may be a dynamic between younger cohorts' more significant emphasis on their mental and emotional self-care and mainstream, or older cohorts' societal responses to their feelings and needs around adulting. It might be that our participants' need to “catch up” on sexual knowledge is as much part of this anxiety-filled social practice of needing “to adult” as the anticipatory stress connected to older adults' reactions to their questions about sex, sexuality and connected practices.

This overarching finding of worries and anxiety around filling the void in knowledge and understanding of sexual health may also be attributed to what researchers in medical education called personality traits of perfectionism (79, 80) linked to students in highly competitive fields where grades, achievement, and educational prestige may impact psycho-emotional health outcomes (81). To many of our participants, their perceptions around this gap or void in their sexual health “funds of knowledge” were often shared with a slight sense of anxiety. Empirical data from studies have looked at the association of mental health states, such as anxiety and depression, to health and medical professions students' grades, clinical competency assessments, and related curriculum completion matters. It might be that for our students, reflecting on these gaps in their SRH knowledge creates similar states of anxiety seen when dealing with similar gaps of knowledge in their course of study. Further study is warranted to understand the processes underlying the relationship between anxiety and knowledge connected to embodied and lived experience of sexuality in the lives of those training to be healers.

We also want to highlight the role of sexual and reproductive health knowledge and practices in the identity-maintaining processes and practices shared in our participants' recollections. For college-enrolled youth, researchers have highlighted the health resources and information available in these settings as connected to identity-building, specifically in how they define sexual wellness (64), an experience and positionality associated with overall health (13). For instance, work by Fraser and colleagues (82) argues that adolescents and emerging adults “engage in extensive and complex processes of research and synthesis to generate their own knowledge base” (82) about sex and sexuality and actively seek out trusted sources elsewhere. In our study, we found respondents questioning not only the knowledge sources and strategies used to learn about sex and reproduction during their childhood and adolescence and how these impacted them as young adults but also reflecting on the impacts of these gaps on their future career choices. Thus, those planning to enter patient-facing careers might also see the need to engage in a similar process as they actively build their knowledge base and repair the fractures. While there is fear that the glut of information on social media may show both facts and fiction about sex, sexuality, and sexual health, we still feel that these moves toward education using personal experience can have positive results. For instance, student-designed and peer-led curricula on sexualities have been received well by others in medical and health training settings, especially those that integrate and center instructors' personal experiences as a pedagogical strategy (83).

### Limitations

There are several limitations to our findings. We interviewed undergraduates preparing for future careers in allied health, medical sciences, or public health, so their responses might have been influenced by their educational goals and the knowledge they have learned as trainees, volunteers, and entry-level employees in patient-facing and other clinical or healthcare settings. Although our sample of interviewees was quite diverse regarding race/ethnicity, most participants self-identified as being cis-gendered/female-assigned at birth. Thus, our research insights cannot be generalized to all college-aged student populations in the US. Our findings might not also apply to research conducted in more traditional campus settings. Our research setting, the campus' unique student health services infrastructure, and its' institutional emphasis on medicine and health care may have influenced our participants' reflections and responses about sexual and reproductive health and their recommendations to increase their knowledge on these issues. In line with recent scholarly work on the experiences of young adults' learning about sex (6, 59, 84), we underline the importance of comprehensive sexual health education in K-12 and post-secondary settings and reccomend that future research assesses the unique needs of cis-gendered men, transgender individuals, and other populations of young adults.

### Implications

Despite the limitations listed above, this study's findings provide a rich account of the effects of past experiences receiving sexual health education from parents, trusted adults, and schools on the professional identities of future healthcare providers. While we cannot change the experience of receiving fractured knowledge and education on sexual and reproductive health for today's professionals, there are ways higher education institutions can intervene and help students “catch up”. In fact, higher education contexts are central to helping college-aged populations understand their bodies and health behaviors. At the university level, implementing a sexual education course as a graduation requirement could create a standardization of information for all young people, not just future healthcare professionals. Our participants comprised a population of young adults planning to enter the medical sciences and healthcare field. The patients that these students will eventually engage with will likely hold concerns about their own sexual and reproductive health and will expect their healthcare providers to display a comprehensive level of sexual health literacy. For those students graduating from other fields, such as social work, they will most likely deal with survivors of sexual assault. Those in education services may find themselves as a middle-school teacher helping a student who may have gotten their first period. Thus, requiring sexual and reproductive health information to be taught at the collegiate level would benefit students regardless of their career aspirations by increasing knowledge about ways to prevent STIs and pregnancy.

Lindberg and Kantor's analysis of a 2011–2019 nationally representative sample of US adolescents found that 43% had received information on delaying sex, using and accessing contraceptives, and preventing STIs and HIV (85). This means that for more than half of the respondents surveyed, access to sexual and reproductive health knowledge was minimal, which most likely barred them from receiving critical information to make informed decisions about their sexual and reproductive lives. In the United States, most states enforce regulations granting parents the ability to have their child “opt-out” of sexual education curriculum (86–88). Thus, a consequence of “opting out” is that education received in the K-12 setting may be a child's only opportunity to learn about their health and safety if it is not discussed at home. While living under their parents' regulations, children only have so much access to information about many topics, including sexual and reproductive health, and the information they find on their own may not be age-, culturally-, or medically-appropriate. Nearly all university students are over eighteen, which reduces the impact of parental rights to make decisions on behalf of their children. By providing a full-length course rather than a module during an already overwhelming university orientation, students previously lacking information about sexual and reproductive health can “catch up” to their peers.

Finally, sexual health literacy learned after K-12 could provide that “baseline” knowledge to young adults living independently for the first time. This knowledge, as they embark towards independence, offers young adults more than just pertinent information on their bodies, sexual health behaviors, and reproductive futures; it allows them to develop new embodied forms of empowerment and self-determination.

## Data Availability

The datasets used in this article are not readily available due to the sensitive nature of the data. Request to access raw qualitative transcripts are in a case by case only and should be directed to amejiame@umn.edu.
